# 

**Published:** 1894-08

**Authors:** 


### CONTENTS.

ORIGINAL ARTICLES—

When May Our Syphilitics Marry. By William S. Gottheil, M.D., New York City ..... 407

Prolapse of Rectum in Child One Year Old. By Ira W. Porter, M.D., Sandford, Fla ..... 410

A Complicated Case of Labor—Placenta Previa—Hydramnios Fatty Degeneration of the Placenta. By V. D. Lockhart, M D., Homer, Ga ..... 411

A Case of Extra-Uterine Pregnancy. By J. G. Earnest, M.D., Atlanta, Ga ..... 414

Operation for Trachoma. By Thomas S. Mitchell, M.D., Columbus, Ga ..... 415

Nasal Sprays. What Apparatus to Use. By George Brown, M.D., Atlanta, Ga ..... 417

SOCIETY REPORTS—

Philadelphia Academy of Surgery ..... 422

BOOK REVIEWS—

An International System of Electro-Therapeutics, for Students, General Practitioners and Specialists. By Horatio R. Bigelow, M.D., and thirty-eight associate Editors ..... 429

Diseases of the Skin. An Outline of the Principles and Practice of Dermatology. By Malcolm Morris, Surgeon to the Skin Department St. Mary's Hospital, London ..... 429

A Manual of Nursing in Pelvic Surgery. By L. S. McMurtry, A.M., M.D ..... 429

SELECTIONS AND ABSTRACTS—
The Treatment of Typhoid Fever ... 430
Tobacco Smoke ... 434
The Modern Treatment of Diphtheria ... 436
Brief Hints on Diseases of the Ear in Children ... 437
Chloral Hydrate—Some of Its Uses ... 440
The Antiseptic Treatment of Gonorrhea ... 442
How Ought We to Treat Gonorrhea Epididymitis ... 445
Catarrhal Urethritis in Boys ... 446
The Treatment of Nephritis ... 447
Trional in Delirium Tremens ... 447
Cauliflower Cancer ... 448
Gonorrheal Infection ... 448

EDITORIAL—
Early Operation for Appendicitis ... 449

EDITORIAL NOTES ... 454

PRESCRIPTION DEPARTMENT ... 456, 457
For Hemorrhagic Diarrhea—For Inhalation—Anti-Rachitic Powder—For Chronic Catarrhal Diarrhea—Diphtheria—Painful Dyspepsia—Headache—Tænia—For Neuralgia.

SPECIAL NOTES ... 458, 460, 462, 464

